# Effects of winter food provisioning on the phenotypes of breeding blue tits

**DOI:** 10.1002/ece3.4048

**Published:** 2018-04-24

**Authors:** Kate E. Plummer, Stuart Bearhop, David I. Leech, Dan E. Chamberlain, Jonathan D. Blount

**Affiliations:** ^1^ Centre for Ecology and Conservation College of Life & Environmental Sciences University of Exeter Penryn Cornwall UK; ^2^ British Trust for Ornithology The Nunnery Thetford Norfolk UK; ^3^ Dipartimento di Scienze della Vita e Biologià dei Sistemi Università degli Studi di Torino Turin Italy

**Keywords:** antioxidant, carotenoid‐based plumage, carry‐over effect, life history trade‐off, oxidative stress, urban

## Abstract

Throughout the Western World, huge numbers of people regularly supply food for wild birds. However, evidence of negative impacts of winter feeding on future reproduction has highlighted a need to improve understanding of the underlying mechanisms shaping avian responses to supplementary food. Here, we test the possibility that carry‐over effects are mediated via their impact on the phenotypes of breeding birds, either by influencing the phenotypic structure of populations through changes in winter survival and/or by more direct effects on the condition of breeding birds. Using a landscape‐scale 3‐year study of blue tits (*Cyanistes caeruleus*), we demonstrate the importance of nutritional composition of supplementary food in determining carry‐over effect outcomes. We show that breeding populations which had access to vitamin E‐rich foods during the previous winter were comprised of individuals with reduced feather carotenoid concentrations, indicative of lower pre‐feeding phenotypic condition, compared to fat‐fed and unfed populations. This suggests that supplementary feeding in winter can result in altered population phenotypic structure at the time of breeding, perhaps by enhancing survival and recruitment of lower quality individuals. However, supplementation of a fat‐rich diet during winter was detrimental to the oxidative state of breeding birds, with these phenotypic differences ultimately found to impact upon reproductive success. Our findings demonstrate the complex nature by which supplementary feeding can influence wild bird populations.

## 1 INTRODUCTION

Where the availability of resources is limited, life history theory predicts that trade‐offs should exist in their allocation toward self‐maintenance and reproduction (Stearns, 1989). For birds, resource trade‐offs can be alleviated by the provisioning of supplementary foods (Davis, Nager, & Furness, 2005; Morosinotto, Villers, Varjonen, & Korpimäki, 2017; Ruffino, Salo, Koivisto, Banks, & Korpimaki, 2014; Simons & Martin, 1990). Food supplementation of wild birds is a prolific and expanding phenomenon, predominantly occurring during winter in gardens and backyards throughout the Western World (Jones & Reynolds, 2008). In the United Kingdom, for example, it has been estimated that 150,000 tonnes of wild bird food are sold annually at a cost of more than £200 million (PFMA, 2015), and in recent years, there has been considerable diversification in the types and the nutritional value of supplementary foods being provisioned (Plummer, Siriwardena, Conway, Risely, & Toms, 2015; Risely & Simm, 2016). However, there is ongoing uncertainty about the ecological impacts of supplementary feeding, and more specifically about the underlying physiological mechanisms shaping avian responses to these abundant anthropogenic food resources.

In a seasonal environment, an individual's ability to resolve physiological trade‐offs can be “carried‐over” to affect future fitness and reproductive investment (Harrison, Blount, Inger, Norris, & Bearhop, 2011). As such, recent research has begun to investigate the consequences of winter supplementary feeding on subsequent avian productivity, reporting both beneficial (Robb et al., 2008) and detrimental effects on breeding performance at the population level (Plummer, Bearhop, Leech, Chamberlain, & Blount, 2013a,b). It has been assumed that such carry‐over effects are mediated via the impacts of winter supplementary food on overwinter survival and/or on avian condition during the breeding season (Brittingham & Temple, 1988; Crates et al., 2016; Gosler, 1996; Plummer et al., 2013b; Robb et al., 2008), although this remains to be studied in detail. Given the enormity of anthropogenic bird feeding activities, and as provisioning of food for wild birds is often advocated as a method for conserving declining populations (Ewen, Walker, Canessa, & Groombridge, 2014; Jones & Reynolds, 2008; RSPB, 2018), it is imperative that the mechanisms driving any negative carry‐over effects of winter feeding are investigated.

One possibility is that negative effects of food supplementation may reflect a change in breeding population structure, such that individuals of relatively poor phenotypic condition are able to survive and recruit to become breeders as a result of winter feeding (Plummer et al., 2013b). Alternatively, dependency on fat‐rich supplementary foods in winter could lead to an unbalanced diet, having a detrimental effect on phenotypic condition and thereby impairing reproductive investment during the subsequent breeding season (Plummer et al., 2013a,b). Phenotypic condition is greatly influenced by oxidative stress, an imbalance between harmful reactive oxygen species (ROS), produced as by‐products of metabolic activity, and the antioxidant defense system (Selman, Blount, Nussey, & Speakman, 2012). As metabolic activity is heightened under harsh environmental conditions (Carrascal, Senar, Mozetich, Uribe, & Domenech, 1998), oxidative stress is likely to pose an increased threat to avian health status during winter. However, sufficient uptake of dietary antioxidants, such as vitamin E and carotenoids, can prevent oxidative stress and consequent biomolecule damage (Selman et al., 2012; Sies & Stahl, 1995). Vitamin E in particular has been shown to play a valuable role in avian ecology (Costantini, 2008), and thus, its increased uptake in winter could potentially enhance phenotypic condition and have overwinter survival benefits. By enabling birds which might otherwise have perished in winter to reach a condition necessary to breed, supplementary feeding with vitamin E has the potential to influence phenotypic variation among breeding birds at the population level.

**Figure 1:**
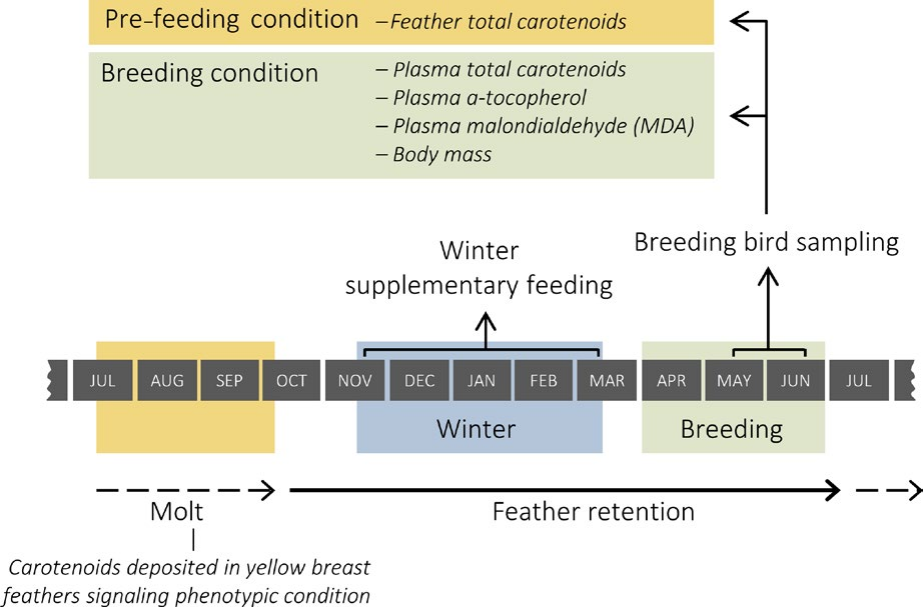
A timeline to illustrate the study's experimental design in relation to the blue tit annual cycle. A novel approach was used to measure the phenotypic condition of birds pre‐winter feeding by measuring condition‐dependent carotenoid concentrations in feathers collected from breeding birds. The experimental protocols were replicated over 3 years

The aim of this study was to test two potential mechanisms mediating carry‐over effects of winter feeding, by examining the effects of winter food supplementation on the average phenotypic condition of breeding blue tit (*Cyanistes caeruleus*) populations. In winter, either fat alone or fat‐plus‐vitamin E was supplemented at a landscape‐scale and compared to unfed controls. Firstly, we predicted that winter vitamin E supplementation would lead to a reduction in the average phenotypic condition of the subsequent breeding population, consistent with the explanation that provisioning may enable individuals in lower condition to recruit. Since it is difficult to follow known individual blue tits across seasons, we used a novel approach to test this prediction by measuring condition‐dependent plumage color. Increased carotenoid deposition in feathers is indicative of an individual's ability to resolve carotenoid allocation trade‐offs between sexual signaling and self‐maintenance and is therefore considered to be a reliable signal of individual phenotypic condition during molt (Møller et al., 2000). In tits, carotenoid‐based yellow plumage has been linked to condition‐dependent survival of adults and has been shown to signal reproductive capacity of both males and females (Doutrelant et al., 2008; Hidalgo‐Garcia, 2006; Hõrak, Ots, Vellau, Spottiswoode, & Møller, 2001; Senar, Figuerola, & Pascual, 2002). Blue tits complete their annual molt in autumn (Morrison et al., 2015), enabling us to use the carotenoid concentration in feathers collected during the breeding season as an index of individual phenotypic condition prior to winter feeding (hereafter, pre‐feeding condition, Figure 1). Secondly, we predicted that winter fat supplementation would lead to a reduction in the average phenotypic condition of breeding birds (hereafter, breeding condition), due to their increased susceptibility to oxidative stress. Breeding condition was measured in terms of body mass and plasma levels of oxidative damage, vitamin E, and carotenoids (Figure 1). Oxidative damage was determined using a biomarker of lipid peroxidation, malondialdehyde (MDA) (Selman et al., 2012), with higher concentrations in the blood reflecting reduced breeding condition. To determine the biological significance of our findings, we also assessed the consequences of individual variation in phenotypic condition measures on breeding performance.

## 2 MATERIALS AND METHODS

### 2.1 Experimental design

The study was conducted over three breeding seasons (2008–2010) in nine deciduous woodlands in Cornwall, UK, averaging 10.7 ha (mean ± 1.1 *SE*) in size and situated at least 2 km apart. The woodlands, which were predominantly comprised of oak (*Quercus* spp.), beech (*Fagus sylvatica*), and sweet chestnut (*Castanea sativa*), were grouped into triplets according to their vegetation composition (see Appendix S1). Within sites, feeders and nest boxes were positioned at an equal density and distribution of *ca*. one feeder and four nest boxes per hectare. See Plummer et al. (2013a) for further detail.

In the first study year, each site within a triplet was randomly allocated to a feeding treatment: (1) unfed, (2) fat only, or (3) fat‐plus‐vitamin E. Treatments were rotated among woodlands within a triplet each year, such that every site received all treatments over the course of the study, and potential confounding effects of year were avoided. Woodlands allocated to one of the fed treatments were provisioned with fresh 150 g fat balls at feeding stations every 10 days during winter to ensure ad libitum availability (Figure 1; 14 December—4 March 2007/08; 18 November—11 March 2008/09 and 2009/10). Fat balls were formulated to tease apart the effects of fats and antioxidants in mediating carry‐over effects, rather than as true replicates of commercial fat balls per se. However, the fat balls do also provide a proxy for low (fat) and high (fat‐plus‐vitamin E) quality supplementary foods widely provided in gardens.

Fat balls were produced using solid vegetable fat (Crisp ‘n Dry; Princes Ltd., Liverpool, UK) and a small amount of yellow food coloring (0.125 ml 100 g^−1^ fat; ASDA Natural Food Colouring, Asda Stores Ltd., Leeds) to increase food attractiveness (Mcgraw, Crino, Medina‐Jerez, & Nolan, 2006). For the fat‐plus‐vitamin E treatment, fat balls were supplemented with 100 mg/kg fat of α‐tocopherol (T3251; Sigma‐Aldrich, Dorset, UK), a concentration equivalent to that occurring in peanuts (Chun, Lee, & Eitenmiller, 2005) which are commonly provisioned to wild birds. All supplements were prepared using standardized methods (Plummer et al., 2013a). Measurements of food use indicated a high level of uptake by the target species, and ring recoveries and stable isotope analysis confirmed that breeding birds had used supplementary foods in winter (Plummer, 2011).

### 2.2 Breeding bird phenotypes and productivity

Nest boxes were inspected every 1–3 days from April to June to determine laying date, brood size changes, and fledging numbers. Body mass (± 0.1 g) and head‐bill length (± 0.05 mm) were measured for all surviving nestlings at 12 days post‐hatching (± 1 day); then, the means were calculated per brood. Fledging success was defined as the proportion of hatched offspring that fledged.

Breeding birds were captured opportunistically on the nest during routine nest checks or using spring traps (Amber Electronics Ltd, Daventry, UK) between day 5 and 18 of the nestling phase (Figure 1; mean nestling age at sampling, 13.06 days ± 0.18 *SE*,* n* = 351). One or both parent blue tits were sampled from 52.63% ± 2.10 (mean ± *SE*) of occupied boxes per site from 2008 to 2010 (total independent nests, *n *=* *243; total individuals, *n *=* *351). Increased reproductive effort resulting from raising a large brood can cause oxidative damage (Wiersma, Selman, Speakman, & Verhulst, 2004), and birds captured later in the nestling phase are likely to have invested more in breeding at the time of sampling than those captured when their nestlings were young. Therefore, a continuous measure of “reproductive effort” was calculated and controlled for in breeding condition analyses, defined as the sum of brood sizes for each nest on each day between the first day post‐hatching and the day of parental sampling (range: 12.0 – 167.0, mean ± *SE*: 78.4 ± 1.6), following Mayfield (1975). As brood size was not recorded daily, in instances of chick mortality, a mean brood size was used for the unknown days from the known values either side. There were no instances of multiple broods.

The sex, age (1 or >1 year), body mass (± 0.1 g), and head‐bill length (± 0.05 mm) of all captured adult birds were recorded, and yellow breast feathers were sampled from a standardized position on the breast. Blood samples (30–60 μl) were collected from the brachial vein of birds captured after day 7 of the nestling phase into an EDTA‐coated capillary tube (Bilbate Ltd., Daventry, UK). Blood samples were centrifuged (4 min; 13,000 ×g) and stored in a cool box, before being returned to the laboratory for plasma extraction and storage at −80°C. Standard methods were used for the quantification of feather total carotenoid concentrations (*n *=* *335), and plasma concentrations of total carotenoids (*n *=* *136), α‐tocopherol (*n *=* *124), and malondialdehyde (MDA; *n *=* *171) (see Appendix S2). Differences in sample sizes among the different biochemical assays are a result of blood sample volume limitations.

### 2.3 Statistical analyses

Statistical analyses were conducted using R version 3.1 (R Core Team, 2014) using linear mixed models (LMMs) with Gaussian errors and backwards stepwise deletions of non‐significant terms (*p *>* *.05). Normality and heteroscedasticity of residuals were checked prior to model simplification, and continuous variables were standardized to enable effect sizes to be compared directly (Gelman, 2008). For *post‐hoc* analyses of feeding treatment differences, ANOVA was used to compare the minimum LMM model with replicate models in which two treatment groups under comparison were paired.

To test the hypothesis that winter supplementary feeding alters the phenotypic structure of breeding populations, we examined the difference in feather total carotenoid concentration between feeding treatment groups by fitting it as a response against treatment. Sex, age, and year were included as covariates, and their two‐way interactions with treatment were tested. As feather carotenoid concentrations were correlated within breeding pairs (Pearson's correlation: *r *=* *.40, *n *=* *108, *p *<* *.001), breeding pair was included in the random term, nested within site, to control for nonindependence. Feather carotenoid concentrations were log‐transformed to reach normality of residuals.

To test for carry‐over effects of winter supplementary feeding on the condition of breeding birds, separate LMMs were applied to plasma α‐tocopherol, total carotenoid, and MDA concentrations and to body mass with feeding treatment fitted as a fixed effect. Feather total carotenoid concentration (log‐transformed) was fitted to test for a relationship between pre‐ and post‐feeding condition, and sex, age, reproductive effort (see above), and year were also included as covariates. Head‐bill length was additionally included as a covariate in body mass analysis to account for skeletal body size variation. Two‐way interactions with treatment were modeled, but inclusion of further two‐ and three‐way interactions was prohibited by overparameterization. A hierarchical nested random effects term—breeding pair nested within box identity nested within site—controlled for data non‐independence. Plasma concentrations of α‐tocopherol and total carotenoids response variables were log‐transformed to reach normality of residuals.

To assess whether phenotypic condition ultimately influenced breeding success, we modeled breeding productivity response variables as a function of either pre‐feeding condition (i.e., feather total carotenoid concentration) or breeding condition (i.e., plasma α‐tocopherol, total carotenoid, and MDA concentrations, or body mass). Winter food supplementation has previously been reported to have a significant negative influence on nestling mass, nestling head‐bill length, and fledging success within these study populations (Plummer et al., 2013b). Therefore, to further help to disentangle carry‐over effects mechanisms, these measures were selected as indicators of breeding productivity in the present analyses. Further, we also assessed whether pre‐feeding condition influenced the onset of breeding, by fitting lay date as a response term against feather total carotenoid concentration only. In all instances, sex and treatment were controlled for as fixed effects and box identity nested within woodland site was included as the random term, fitted using LMMs. Nestling age at sampling was additionally controlled in the analyses using nestling mass or head‐bill length as the response term. Fledging success models were fitted using a binomial error distribution. The effects of each phenotypic condition measure were modeled separately to maximize sample size, as we did not have data for all measures from all individuals. However, the outcomes of the analyses were largely unchanged when using a reduced sample and fitting all breeding condition measures as predictors within a single model (see Appendix S3, Table S4). Data are reported as means ± 1 *SE*.

## 3 RESULTS

### 3.1 Breeding population structure

**Figure 2:**
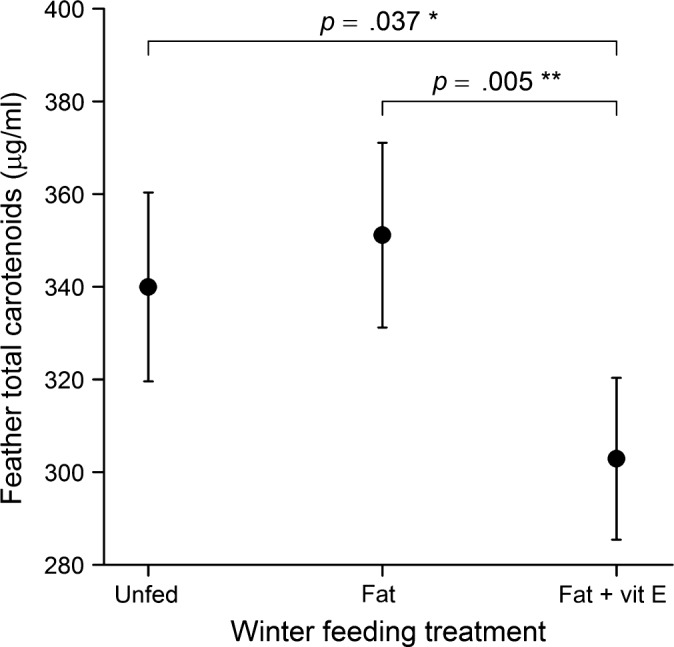
Total carotenoid concentrations in the feathers of breeding blue tits in relation to winter feeding treatment. Mean (±*SE*) values predicted from the GLMM minimum model are shown, plus significant *post‐hoc* pairwise comparisons

Feather total carotenoid concentrations, which reflect condition prior to the onset of the previous winter's food supplementation (Figure 1), differed significantly among winter feeding treatment groups (*n *=* *335, $\chi_{2}^{2}$ = 8.52, *p *=* *.014; Appendix S3). Birds in the fat‐plus‐vitamin E group had lower concentrations of carotenoids in their feathers compared to control and fat‐fed birds (Figure 2). Feather carotenoids also differed among years ($\chi_{2}^{2}$ = 16.76, *p *<* *.001), and males had marginally higher levels than females, although this was not statistically significant ($\chi_{2}^{2}$ = 3.23, *p *=* *.07). However, there were no significant interactions between treatment and either sex, age, or year (*p *>* *.42).

### 3.2 Breeding condition

**Figure 3:**
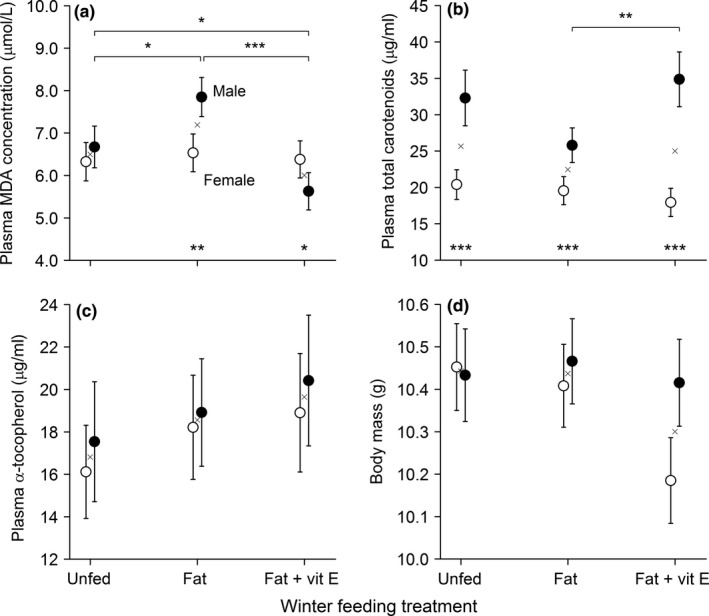
The effect of winter feeding treatment on phenotypic condition of breeding male (filled) and female (open) blue tits, in the form of plasma concentrations of (a) malondialdehyde (MDA), (b) total carotenoid, (c) α‐tocopherol, and (d) body mass. Mean (±*SE*) values are predicted from the GLMM minimum model, with sex × treatment interaction reintroduced where not significant (c, d). Feeding treatment means are marked by a cross. *Post‐hoc* pairwise significance of sex differences within treatments is reported at the bottom of each panel, and differences in male values between treatments are reported at the top, where *p <* *.05; **p < .01, and ***p < .001

Winter supplementary feeding had a significant effect on the concentrations of MDA and total carotenoids in the plasma of breeding blue tits (Table 1; Appendix S3). MDA concentrations during the breeding season were significantly higher following fat supplementation in winter, compared to those birds which had received fat‐plus‐vitamin E (*n *=* *165, $\chi_{2}^{2}$ = 9.64, *p *=* *.008), while neither treatment was significantly different from the unfed control group (*p *>* *.15). MDA concentrations also differed between the sexes (Table 1, Figure 3a). Within the unfed group, plasma MDA concentrations were not significantly different between males and females (*p *=* *.42), but fat‐fed males had significantly greater MDA concentrations than both females from the same group and unfed males (Figure 3a). Conversely, vitamin E‐fed males had significantly lower MDA concentrations than both females in the same treatment group and unfed males (Figure 3a). Female MDA concentrations were not significantly different between treatments (*p *>* *.25).

**Table 1:** General linear mixed models to assess the condition of breeding birds in relation to winter feeding, pre‐feeding condition (feather total carotenoid concentration), and breeding effort. All significant terms within each minimum model are reported plus non‐significant main effects, following stepwise deletion. Estimates for plasma total carotenoid and α‐tocopherol concentrations are on a log scale. Significant terms in bold

Fixed effect	Plasma MDA			Plasma α‐tocopherol			Plasma total carotenoids			Body mass		
	χ^2^	*df*	*p*	χ^2^	*df*	*p*	χ^2^	*df*	*p*	χ^2^	*df*	*p*
Treatment	**8.04**	**2**	**.018**	2.52	2	.284	2.64	2	.267	3.71	2	.157
Sex	0.94	1	.332	0.40	1	.528	**37.09**	**1**	**<.001**	2.55	1	.110
Age	0.59	1	.441	**10.55**	**1**	**.001**	**5.98**	**1**	**.015**	**4.31**	**1**	**.038**
Head‐bill length	–	–	–	–	–	–	–	–	–	**59.31**	**1**	**<.001**
Feather carotenoids	0.67	1	.414	0.75	1	.386	0.46		.496	0.33	1	.567
Reproductive effort	0.09	1	.762	0.00	1	.957	**4.14**	**1**	**.042**	**40.00**	**1**	**<.001**
Year	**104.40**	**2**	**<.001**	**41.36**	**2**	**<.001**	3.38	2	.185	**33.78**	**2**	**<.001**
Treatment × sex	**13.42**	**2**	**.001**	–	–	–	**6.92**	**2**	**.031**	–	–	–

Winter supplementary feeding also significantly influenced the plasma total carotenoid concentrations of males, but not females (Table 1; Figure 3b). The direction of the effect mirrored that for plasma MDA, with males in the fat‐plus‐vitamin E group having significantly higher concentrations of plasma carotenoids than males provisioned with fat alone (*p *=* *.010; Figure 3b).

Concentrations of α‐tocopherol in the plasma varied considerably among individuals (23.13 ± 1.06 μg/ml, range: 3.30–70.91 μg/ml), and body mass was strongly negatively correlated with reproductive effort (*p *<* *.001; Table 1), but neither were influenced by winter feeding treatment (*p *≥* *.16; Table 1; Figure 3c and d). Increased reproductive effort also led to a reduction in total carotenoids in the plasma (*p *=* *.042; Table 1). All measures of breeding condition showed significant differences among individuals of different ages and/or as a result of annual variation (*p *≤* *.038; Table 1), although there was no evidence that these relationships differed between treatment groups (*p *≥* *.10). No significant relationships were found between pre‐feeding condition (i.e., feather carotenoid concentration, *p *≥* *.39) and measures of breeding condition, irrespective of feeding treatment (*p *≥* *.24; Table 1).

### 3.3 Breeding performance

**Figure 4:**
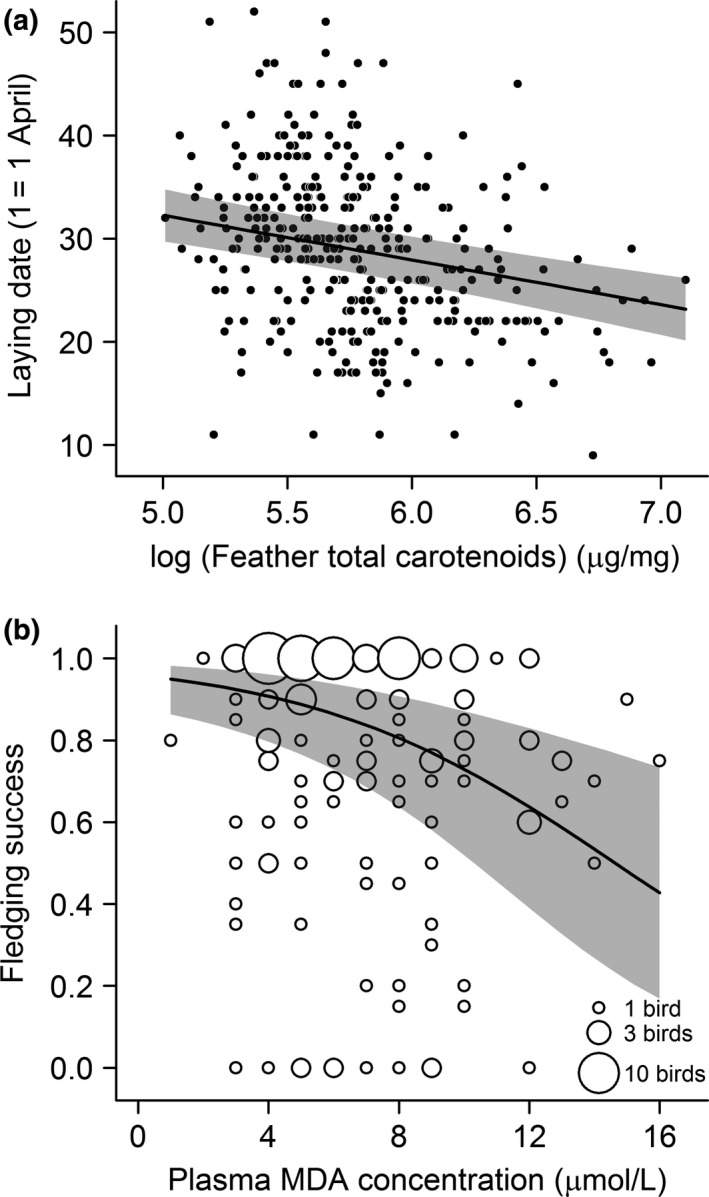
The effect of individual variation in phenotypic condition on breeding performance. (a) Laying date in relation to feather total carotenoid concentration. (b) Fledging success in relation in plasma malondialdehyde (MDA) concentration. Data distribution has been illustrated by rounding MDA values to 1 μmol/L and fledging success to 0.05, then scaling point areas by bird frequencies with examples given in the legend. Lines and 95% confidence intervals (shaded gray) are predicted from the minimum models

Having controlled for the effects of treatment within the analyses, individuals with greater concentrations of carotenoids in their feathers were found to begin egg laying significantly earlier than those with fewer feather carotenoids ($\chi_{1}^{2}$ = 29.52, *p *<* *.001; Appendix S3; Figure 4a) and produced offspring that were smaller ($\chi_{1}^{2}$ = 7.22, *p *=* *.007). However, feather carotenoid concentrations did not significantly influence other later stages of the breeding season, including mean offspring mass (*p *=* *.08) and fledging success (*p *=* *.74). By contrast, individuals suffering greater oxidative damage, in the form of higher plasma concentrations of MDA, produced offspring which were structurally smaller ($\chi_{1}^{2}$ = 12.74, *p *<* *.001) and had reduced fledging success ($\chi_{1}^{2}$ = 13.07, *p *<* *.001; Figure 4b). Birds with increased body mass also fledged fewer ($\chi_{1}^{2}$ = 8.10, *p *=* *.004), but larger ($\chi_{1}^{2}$ = 5.72, *p *=* *.016), young. However, offspring mass was not significantly influenced by the concentrations of MDA, α‐tocopherol, or total carotenoids in the plasma of breeding adults (*p *>* *.15). Further, concentrations of carotenoids and α‐tocopherol in the plasma did not significantly affect any measures of breeding performance (*p *>* *.23).

## 4 DISCUSSION

Recent research has reported that supplementary feeding during winter may have negative impacts on the future breeding performance of birds at the population level, but the mechanisms driving these negative effects have been uncertain. Our results support the hypothesis that winter food supplementation both alters the phenotypic structure of breeding populations and also influences the condition of breeding birds. Importantly, the nature of these responses depends on the nutritional composition of supplemented foods. While supplementation with a combination of vitamin E and fat appears to have improved the survival, recruitment and breeding condition of birds which were in significantly poorer condition prior to feeding, provisioning with fat alone had detrimental effects on the condition of breeding birds. Further, we show that reductions in individual condition can ultimately have adverse effects on breeding performance. Our findings suggest that negative effects of winter food supplementation are mediated via impacts on overwinter survival and on the condition of individuals during the breeding season.

Carotenoids can have limited environmental availability, resulting in physiological trade‐offs in their allocation between self‐maintenance and sexually selected coloration (Mcgraw & Ardia, 2003). We found that at the population level, blue tits breeding in woodlands which had been supplied with fat‐plus‐vitamin E in the previous winter had lower levels of carotenoids in their breast feathers than those which were unfed or were fed with fat alone. As molt is completed annually between the breeding season and the beginning of winter (Morrison et al., 2015), this finding suggests that supplementation of vitamin E in winter altered the survival and recruitment prospects of individuals.

Carotenoid‐based signals are considered to be a reliable indicator of individual condition at the time of feather molt (Møller et al., 2000; Olson & Owens, 1998), with numerous studies linking phenotypic condition indices and carotenoid‐based coloration in birds (Harper, 1999; Mougeot et al., 2010; Peters, Kurvers, Roberts, & Delhey, 2011). In tits, yellow breast feather coloration has also been linked to condition‐dependent survival and reproductive output (Doutrelant et al., 2008; Hidalgo‐Garcia, 2006; Hõrak et al., 2001). Indeed, we also report evidence that individuals with greater carotenoid levels in the feathers initiated breeding earlier, a notable precursor for reproductive success (Nilsson, 2000). Increased circulating vitamin E acquired from winter feeders may have enhanced antioxidant defenses and/or immunity, therefore, improving individual health status (Surai, 2007). As a consequence, winter vitamin E supplements could have enabled greater overwinter survival of individuals in poor phenotypic condition and facilitated their recruitment into breeding populations. We lack specific data on winter survival due to logistical constraints in tracking individual blue tits across seasons within our study populations. However, as this study was conducted over multiple sites and years, it is highly implausible that the reduced mean concentrations of carotenoids observed in the feathers of vitamin E‐fed populations could have occurred by chance, and it is also unlikely that they result from carry‐over effects of treatments from previous winters. If this were the case, one would have expected to find no difference in feather carotenoids between treatment groups in the first breeding season, as carotenoids were deposited in feathers prior to any food supplementation taking place. However, our results showed that the difference in feather carotenoid concentrations between treatment groups was consistent across years. Changes to the phenotypic structure of breeding populations as a consequence of winter food supplementation could result in an apparent reduction in productivity at a population level, if a greater number of individuals with relatively low reproductive capacity are able to breed (Plummer et al., 2013b). Alternatively, it is also possible that enhanced survival could result in density‐dependent influences on population‐level productivity, due to increased competition for resources in the breeding season.

The consequences of winter food supplementation were also found to be carried‐over and to influence the condition of birds in the subsequent breeding season, which in turn impacted on their overall reproductive success. For many bird species, the brood‐rearing phase represents a time of intense energetic demand (Bryant & Tatner, 1991). Elevated parental effort during this period has been shown to impose high costs on current condition and subsequent survival (Hõrak, 1995; Nur, 1984), and oxidative stress is likely to be at least partly responsible for these observed reproductive costs (Alonso‐Alvarez et al., 2004; Blount, Vitikainen, Stott, & Cant, 2016; Nilsson, 2002; Wiersma et al., 2004). In the present study, we used malondialdehyde (MDA) concentration in the plasma as a marker of oxidative damage to lipids during brood rearing and presented evidence that elevated plasma MDA can lead to a reduction in the number of offspring an individual can successfully fledge. We found that susceptibility to oxidative damage was significantly reduced in birds provisioned with vitamin E several weeks or months earlier, yet it was not correlated with prebreeding condition. A probable explanation for the combined results, therefore, is that previously “poor‐quality” individuals were able to survive and breed as a result of vitamin E supplementation, and their condition was sufficiently enhanced by feeding that they then exhibited better phenotypic condition compared to fat‐fed and control birds in the breeding season.

Interestingly, the variation in plasma MDA concentrations observed between feeding treatments was entirely driven by a marked difference in the oxidative damage responses of males and females. Concentrations of carotenoids in the plasma, which form part of the antioxidant defense system and may contribute to combating oxidative stress (but see Costantini & Møller, 2008), also showed the same differential sex‐based response. The concentrations of MDA and total carotenoids in the plasma of females did not differ significantly between fed and unfed birds. By comparison, males supplemented with fat‐plus‐vitamin E in winter showed lower oxidative damage, while the opposite was true of males supplemented with fat alone.

The differences in the susceptibility of males and females to oxidative stress could be indicative of sex‐based variation in antioxidant uptake, storage, and mobilization. For example, males are considered to be more dominant at bird feeders and have a greater uptake of winter supplementary food than females (Hegner, 1985; Robb et al., 2011). Therefore, males using antioxidant‐rich supplementary foods could benefit from greater condition‐based benefits than females, enabling them to resolve physiological trade‐offs more effectively during brood rearing. By comparison, the high uptake of polyunsaturated fat by males in the fat‐only treatment group could render them more susceptible to oxidative stress. Alternatively, it might not simply be the case that winter feeding predominantly affects males, but instead that our findings also reflect differential reproductive roles between the sexes across the breeding season. More specifically, the carry‐over effects of winter food supplementation on female blue tits may be better approximated during the egg‐laying phase of reproduction, when they adopt the majority of the reproductive work load (Perrins, 1979). Indeed, winter vitamin E supplementation has previously been linked to increased investment in egg production (Plummer et al., 2013a). As such, differences in maternal condition between treatment groups may become difficult to distinguish during brood‐rearing. By comparison, as males have not made a substantial direct investment into offspring fitness (c.f. egg production) until brood provisioning, the benefits of vitamin E supplementation for condition still prevail at this later stage. We also note that benefits to health status reported during the brood‐rearing period are unlikely to reflect a direct investment of vitamin E acquired at winter feeders into antioxidant defense (Mcgraw & Toomey, 2010; Negro, Figuerola, Garrido, & Green, 2001), as there was no evidence vitamin E supplementation affected concentrations of vitamin E circulating in the plasma.

Although provisioning of supplementary food has largely been considered to be beneficial to garden birds, the evidence that winter supplementary feeding can be detrimental to breeding success has highlighted a lack of understanding about the true impacts of large‐scale supplementation upon wild populations (Harrison et al., 2010; Plummer et al., 2013a,b). The findings of this study highlight some potential mechanisms by which impacts of winter feeding may be carried‐over to influence events in the subsequent breeding season. To our knowledge, this study provides the first evidence of a carry‐over effect of winter food supplementation on the phenotypic condition of wild birds during breeding. The findings also strongly suggest that winter supplementary feeding can alter the phenotypic structure of wild bird populations. The provision of vitamin E appears to have helped to alleviate physiological trade‐offs, such that individuals in poor phenotypic condition in the lead up to winter had improved survival and/or a capacity to reach a condition threshold necessary to reproduce during the subsequent breeding season. Furthermore, the opposing impacts of provisioning fat alone compared to fat‐plus‐vitamin E on physiological condition suggest that negative carry‐over effects of winter feeding may be the consequence of a fat‐rich, unbalanced diet, as has previously been hypothesized (Plummer et al., 2013a,b). This is particularly relevant given the substantial variation that exists in commercially available fat products; from high‐quality foods enriched with antioxidant‐rich nuts and seeds, to low‐quality foods bulked out with nutrient‐poor fillers such as wheat husks or ash. These findings, therefore, stress the importance of considering the nutritional composition of supplementary food sources and suggest that more research is required to determine how supplementary feeding may contribute to population size trajectories.

## CONFLICT OF INTEREST

None declared.

## DATA ACCESSIBILITY

Data for this article are available on the Dryad Digital Repository, https://doi.org/10.5061/dryad.92jq2km (Plummer, Bearhop, Leech, Chamberlain, & Blount, 2018).

## AUTHOR CONTRIBUTIONS

KEP, SB, DIL, DEC, and JDB designed the research. KEP collected and analyzed the data. KEP and JDB wrote the first draft, and all authors approved the manuscript for publication.

## ETHICAL APPROVAL

All applicable institutional and/or national guidelines for the care and use of animals were followed. Tissue samples were collected under Home Office license (PIL 30/8161).
