# Supplementary material for: Effects of winter food provisioning on the phenotypes of breeding blue tits
Source: Ecol Evol. 2018 Apr 24;8(10):5059–68. doi: 10.1002/ece3.4048 (PMC5980576; doi:10.1002/ece3.4048)
Supplement: Supplementary file 1 [file ECE3-8-5059-s001.pdf]

Supplementary material

Effects of winter food provisioning on the phenotypes of breeding blue tits

Kate E. Plummer, Stuart Bearhop, David I. Leech, Dan E. Chamberlain & Jonathan D. Blount

Supporting Information

Appendix S1. Description of woodland sites.

Appendix S2. Supporting methods for biochemical assays.

Appendix S3. Model summary tables for all analyses presented in the results.

Appendix S1

Table S1 | Description of nine deciduous woodlands in Cornwall, UK, surveyed in breeding seasons 2008 - 2010. The numbers of nest boxes per site, as well as their mean occupancy rate over the 3 years of the study is shown. 'Woodland features' are classified low (*) to high (***), (see Plummer 2011 for further detail). For vegetation composition, species are listed in order of prevalence.

			Woodland features				Vegetation composition		
Site	No. boxes	Mean occupancy (% ± SE)	Size (hec)	Periphery woods	Settlement proximity	Public access	Trees	Understorey	Ground cover
Woodland triplet 1									
1-A	40	47.5 ± 3.8	16.7	**	***	**	Beech, oak, sweet chestnut, sycamore	Holly, hazel	Ivy, bryophytes, ferns, brambles, bluebells
1-B	44	62.5 ± 0.0	14.2	***	**	**	Oak, sweet chestnut	Holly, hazel	Brambles, honeysuckle, bryophytes, ivy
1-C	39	26.7 ± 4.4	10.0	**	*	***	Oak, beech, ash, sweet chestnut	Holly, hazel	Brambles, ferns, ivy, bryophytes, honeysuckle
Woodland triplet 2									
2-A	43	54.2 ± 3.0	13.1	**	***	*	Oak (coppice)	Hazel, holly	Brambles, sedges, honeysuckle
2-B	32	40.0 ± 3.8	6.6	**	***	*	Oak (coppice), beech, sycamore	Hazel, holly	Brambles, ivy, bluebells
2-C	41	68.3 ± 3.6	9.5	*	**	***	Oak (coppice), beech, birch	Hazel, holly	Honeysuckle, brambles, bluebells, bryophytes
Woodland triplet 3									
3-A	34	33.3 ± 6.5	9.3	**	*	*	Beech, ash, sycamore, oak	Laurel, holly	Ivy, bryophytes, ferns, bluebells
3-B	39	30.0 ± 3.8	7.6	***	*	***	Ash, sycamore, beech, oak	Holly, hazel	Ivy, brambles, bryophytes
3-C	34	31.7 ± 3.0	9.4	**	*	*	Oak, beech, sycamore	Rhododendron	Bryophytes, brambles

References

Plummer KE (2011) The effects of over-winter dietary provisioning on health and productivity of garden birds. Ph.D., University of Exeter.

Appendix S2

Description of the methods used to quantify feather carotenoid concentrations and concentrations of carotenoids, α-tocopherol and malondialdehyde in the plasma for adult birds.

Measurement of feather total carotenoid concentration

Mechanical extraction was used to isolate carotenoid pigments from feathers (Stradi *et al.*, 1995). Whole feathers were washed separately in ethanol (30 s) and hexane (30 s), then blotted dry to remove surface lipids. 3 – 5 mg of yellow barbules were trimmed and ground for 15 minutes at 30 Hz in the presence of 2ml methanol, using a Retsch MM200 micronizer equipped with zirconium oxide grinding jar and balls (Retsch UK Ltd., Castleford, UK). Samples were filtered by injection through a Sep-Pak Light C_{18} Cartridge (Waters Ltd., Dublin), and their absorbance measured at 450nm using a spectrophotometer (Nicolet Evolution 500).

Measurement of plasma total carotenoid and α-tocopherol concentrations

To measure concentrations of antioxidants in the blood, a 10µl aliquot of plasma was mixed with 10µl of 5% sodium chloride and 20µl ethanol, then vortexed for 20 seconds. 600µl of hexane was added and the mixture vortexed for a further 20 seconds, then centrifuged for 4 minutes (13,000 × g). The hexane phase was drawn off and total carotenoid concentration determined by spectrophotometry at 450nm (Nicolet Evolution 500; Thermo Electron Corp., Hemel Hemstead, U.K.), using the extinction coefficient of lutein in hexane (Craft & Soares, 1992). For determination of α-tocopherol concentrations, 500µl of hexane extract was dried and the residue re-dissolved in 150µl methanol, then injected (60µl) into a high-performance liquid chromatography system (HPLC; Dionex Corporation, California, USA). Separation utilised a 3µ C_{18} reverse-phase column (15 cm x 4.6 mm) (Spherisorb S30DS2; Phase separations, Clwyd, UK), with a mobile phase of MeOH:water (97:3 v/v) at a flow rate of 1.1mL min^{-1}. Fluorescence detection (Dionex RF2000) was performed at 295nm (excitation) and 330nm (emission). The α-tocopherol peak was identified and quantified by comparison with a standard solution of α-tocopherol (T3251 Sigma-Altrich) in methanol.

Measurement of plasma malondialdehyde concentration

Plasma concentrations of malondialdehyde (MDA) were determined by HPLC (Agarwal & Chase, 2002, Blount & Pike, 2012). A 10µl aliquot of plasma was vortex mixed with 10µl of butylated hydroxytoluene (BHT) (0.05% w/v in 95% ethanol), 80µl of phosphoric acid (0.44 M) solution and 20µl of 2-thiobarbituric acid (TBA) (42 mM), then heated at 100°C for 1 hour in a dry bath incubator. After cooling on ice (5 min), 50µl of *n*-butanol was added and the mixture vortexed for 20s, then centrifuged for 3 minutes at 4°C (13,000 × g). The upper phase, containing the MDA-TBA adduct, was recovered and 20µl injected into an HPLC system (Dionex Corporation, California, USA) fitted with a Hewlett-Packard Hypersil 5µ ODS 100 x 4.6 mm column and a 5µ ODS guard column, maintained at 37°C. A mobile phase of methanol-buffer (40:60 v/v) was run at a flow rate of 1ml min^{-1}; using a buffer of 50mM potassium monobasic phosphate adjusted to pH 6.8 using 5M potassium hydroxide. Fluorescence detection (Dionex RF2000) was performed at 515 nm (excitation) and 553 nm (emission). Plasma MDA concentrations were calibrated using standards of 1,1,3,3-tetraethoxypropane (TEP) serially diluted with 40% ethanol in a parallel assay.

References

Agarwal R, Chase SD (2002) Rapid, fluorimetric-liquid chromatographic determination of malondialdehyde in biological samples. Journal of Chromatography B-Analytical Technologies in the Biomedical and Life Sciences, **775**, 121-126.

Blount JD, Pike TW (2012) Deleterious effects of light exposure on immunity and sexual coloration in birds. Functional Ecology, **26**, 37-45.

Craft NE, Soares JH (1992) Relative solubility, stability, and absorptivity of lutein and β-carotene in organic solvents. Journal of Agricultural and Food Chemistry, **40**, 431-434.

Stradi R, Celentano G, Nava D (1995) Separation and identification of carotenoids in birds plumage by high-performance liquid-chromatography diode-array detection. Journal of Chromatography B-Biomedical Applications, **670**, 337-348.

Appendix S3

Table S1 | Summary of general linear mixed models used to examine the differences in blue tit phenotypes between winter food supplementation treatment groups. The dependent variables were measures of pre-feeding condition (feather total carotenoids) and breeding condition (plasma α-tocopherol, total carotenoid and MDA concentrations or body mass). All fitted main effects are shown along with their level of significance. Models also included all two-way interactions between 'treatment' and the other covariates, but only the significant interaction terms are shown. The sample size (n) for each model is also shown.

Dependent variable	Fixed effect	χ^2	*d.f.*	*p*	
Pre-feeding condition					
Feather total carotenoids	Treatment	8.52	2	0.014	*
(n = 335)	Sex	3.23	1	0.072	
	Age	0.05	1	0.832	
	Year	16.76	2	< 0.001	***
Breeding condition					
Plasma MDA	Treatment	8.04	2	0.018	*
(n = 165)	Sex	0.94	1	0.332	
	Age	0.59	1	0.441	
	Feather carotenoids	0.67	1	0.414	
	Reproductive effort	0.09	1	0.762	
	Year	104.40	2	< 0.001	***
	Treatment × sex	13.42	2	0.001	***
Plasma α-tocopherol	Treatment	2.52	2	0.284	
(n = 121)	Sex	0.40	1	0.528	
	Age	10.55	1	0.001	***
	Feather carotenoids	0.75	1	0.386	
	Reproductive effort	0.00	1	0.957	
	Year	41.36	2	< 0.001	***
Plasma total carotenoids	Treatment	2.64	2	0.267	
(n = 132)	Sex	37.09	1	< 0.001	***
	Age	5.98	1	0.015	*
	Feather carotenoids	0.46	0	0.496	
	Reproductive effort	4.14	1	0.042	*
	Year	3.38	2	0.185	
	Treatment × sex	6.92	2	0.031	*
Body mass	Treatment	3.71	2	0.157	
(n = 334)	Sex	2.55	1	0.110	
	Age	4.31	1	0.038	*
	Head – bill length	59.31	1	< 0.001	***
	Feather carotenoids	0.33	1	0.567	
	Reproductive effort	40.00	1	< 0.001	***
	Year	33.78	2	< 0.001	***

Table S2 | Summary of general and generalised linear mixed models used to examine the effect of pre-feeding condition (i.e. feather total carotenoid concentration) on breeding performance. The dependent variables were the onset of breeding (lay date) and three measures of breeding performance previously shown to respond negatively to winter feeding at a population level (nestling mass, nestling head-bill length, fledgling success, see Plummer *et al.* 2013). All fitted terms are shown along with their level of significance. The sample size (n) is also shown.

Dependent variable	Fixed effect	χ^2	*d.f.*	*p*	
Effect of pre-feeding condition on breeding performance (n = 335)					
Lay date	Feather carotenoids	29.52	1	< 0.001	***
(n = 335)	Sex	0.32	1	0.573	
	Treatment	3.24	2	0.198	***
Nestling mass	Feather carotenoids	3.02	1	0.082	
(n = 312)	Nestling age	19.07	1	< 0.001	***
	Sex	1.43	1	0.231	
	Treatment	2.57	2	0.277	
Nestling head-bill length	Feather carotenoids	7.22	1	0.007	**
(n = 313)	Nestling age	67.57	1	< 0.001	***
	Sex	4.55	1	0.033	*
	Treatment	6.42	2	0.040	*
Fledging success	Feather carotenoids	0.11	1	0.741	
(n = 335)	Sex	3.64	2	0.057	
	Treatment	3.15	3	0.207	

Table S3 | Summary of general and generalised linear mixed models used to examine the effect of different breeding condition measures (i.e. plasma α-tocopherol, total carotenoid and MDA concentrations or body mass) on breeding performance. The dependent variables were three measures of breeding performance previously shown to respond negatively to winter feeding at a population level (nestling mass, nestling head-bill length, fledgling success, see Plummer *et al.* 2013). All fitted terms are shown along with their level of significance. The sample size (n) for each model is also shown.

Dependent variable	Fixed effect	χ^2	*d.f.*	*p*	
Effect of plasma MDA on breeding performance (n = 162)					
Nestling mass	Plasma MDA	2.47	1	0.116	
(n = 162)	Nestling age	16.55	1	< 0.001	***
	Sex	2.01	1	0.157	
	Treatment	2.22	2	0.330	
Nestling head-bill length	Plasma MDA	12.74	1	< 0.001	***
(n = 162)	Nestling age	42.80	1	< 0.001	***
	Sex	2.96	1	0.086	
	Treatment	4.74	2	0.094	
Fledging success	Plasma MDA	13.07	1	< 0.001	***
(n = 171)	Sex	1.59	1	0.207	
	Treatment	1.51	2	0.469	
Effect of plasma α-tocopherol on breeding performance					
Nestling mass	Plasma α-tocopherol	8.59	1	0.958	
(n = 116)	Nestling age	0.14	1	0.003	**
	Sex	5.43	1	0.703	
	Treatment	8.59	2	0.065	
Nestling head-bill length	Plasma α-tocopherol	1.47	1	0.225	
(n = 116)	Nestling age	11.91	1	< 0.001	***
	Sex	0.48	1	0.486	
	Treatment	6.80	2	0.033	*
Fledging success	Plasma α-tocopherol	0.05	1	0.815	
(n = 124)	Sex	0.29	1	0.588	
	Treatment	0.44	2	0.802	
Effect of plasma total carotenoids on breeding performance					
Nestling mass	Plasma total carotenoids	0.16	1	0.689	
(n = 128)	Nestling age	6.19	1	0.013	*
	Sex	0.00	1	0.945	
	Treatment	8.59	2	0.014	*
Nestling head-bill length	Plasma total carotenoids	0.27	1	0.604	
(n = 128)	Nestling age	14.95	1	< 0.001	***
	Sex	0.44	1	0.508	
	Treatment	6.20	2	0.045	*
Fledging success	Plasma total carotenoids	0.28	1	0.599	
(n = 136)	Sex	0.14	1	0.710	
	Treatment	1.07	2	0.585	

Table S3 ***con'd***

Dependent variable	Fixed effect	χ^2	*d.f.*	*p*	
Effect of body mass on breeding performance					
Nestling mass	Body mass	5.72	1	0.016	*
(n = 326)	Nestling age	22.95	1	< 0.001	***
	Sex	0.42	1	0.518	
	Treatment	7.99	2	0.018	*
Nestling head-bill length	Body mass	1.91	1	0.167	
(n = 327)	Nestling age	68.22	1	< 0.001	***
	Sex	2.45	1	0.117	
	Treatment	10.73	2	0.004	**
Fledging success	Body mass	8.10	1	0.004	**
(n = 350)	Sex	6.78	1	0.009	**
	Treatment	2.70	2	0.259	

Table S4 | Summary of additional general and generalised linear mixed models used to simultaneously examine the effect of different breeding condition measures (i.e. plasma α-tocopherol, total carotenoid and MDA concentrations, and body mass) on breeding performance using a reduced sample of individuals. There was no evidence of collinearity among breeding condition predictor terms ($r \leq 0.44$). The dependent variables are as above (Table S3). All fitted terms are shown along with their level of significance. The sample size (n) for each model is also shown.

Dependent variable	Fixed effect	χ^2	*d.f.*	*p*	
Effect of breeding condition on breeding performance					
Nestling mass	Plasma MDA	3.06	1	0.080	
(n = 83)	Plasma α-tocopherol	0.50	1	0.478	
	Plasma total carotenoids	3.37	1	0.067	
	Body mass	0.65	1	0.421	
	Nestling age	23.15	1	< 0.001	***
	Sex	2.28	1	0.131	
	Treatment	1.42	2	0.491	
Nestling head-bill length	Plasma MDA	4.21	1	0.040	*
(n = 83)	Plasma α-tocopherol	0.50	1	0.654	
	Plasma total carotenoids	0.11	1	0.745	
	Body mass	0.01	1	0.906	
	Nestling age	22.58	1	< 0.001	***
	Sex	2.46	1	0.117	
	Treatment	2.87	2	0.238	
Fledging success	Plasma MDA	5.42	1	0.020	*
(n = 88)	Plasma α-tocopherol	0.20	1	0.653	
	Plasma total carotenoids	2.76	1	0.097	
	Body mass	0.89	1	0.344	
	Sex	2.47	1	0.116	
	Treatment	0.57	2	0.751	

References

Plummer KE, Bearhop S, Leech DI, Chamberlain DE, Blount JD (2013) Winter food provisioning reduces future breeding performance in a wild bird. *Scientific Reports*, **3**.
